# Dissolution-Induced Nanowire Synthesis on Hot-Dip Galvanized Surface in Supercritical Carbon Dioxide

**DOI:** 10.3390/nano7070181

**Published:** 2017-07-11

**Authors:** Aaretti Kaleva, Ville Saarimaa, Saara Heinonen, Juha-Pekka Nikkanen, Antti Markkula, Pasi Väisänen, Erkki Levänen

**Affiliations:** 1 Laboratory of Materials Science, Tampere University of Technology, P.O. Box 589, FI-33101 Tampere, Finland; saara.heinonen@tut.fi (S.H.); juha-pekka.nikkanen@tut.fi (J.-P.N.); erkki.levanen@tut.fi (E.L.); 2 Top Analytica Oy, Ruukinkatu 4, FI-20540 Turku, Finland; ville.saarimaa@topanalytica.com; 3 SSAB Europe Oy, Harvialantie 420, FI-13300 Hämeenlinna, Finland; antti.markkula@ssab.com (A.M.); pasi.vaisanen@ssab.com (P.V.)

**Keywords:** zinc hydroxycarbonate, nanowire, supercritical carbon dioxide

## Abstract

In this study, we demonstrate a rapid treatment method for producing a needle-like nanowire structure on a hot-dip galvanized sheet at a temperature of 50 °C. The processing method involved only supercritical carbon dioxide and water to induce a reaction on the zinc surface, which resulted in growth of zinc hydroxycarbonate nanowires into flower-like shapes. This artificial patina nanostructure predicts high surface area and offers interesting opportunities for its use in industrial high-end applications. The nanowires can significantly improve paint adhesion and promote electrochemical stability for organic coatings, or be converted to ZnO nanostructures by calcining to be used in various semiconductor applications.

## 1. Introduction

Hot-dip galvanized (HDG) coatings are widely used in automotive and construction industries. These zinc coatings are most often the outermost material surfaces, which is why they require sufficient corrosion protection and good aesthetics [1]. Painting is the most common method for improving the aforementioned properties. However, the galvanized surface usually needs additional surface modification before painting, e.g., chemical pretreatment, to provide satisfactory adhesion to the substrate [2]. Weathering of a HDG surface means deliberately corroding the surface to produce a patina layer that contains dissolution products of zinc. This patina layer hinders the corrosion rate of the material and enhances paint adhesion [3,4,5]. In atmospheric corrosion, formation of the patina layer on HDG coating starts by zinc reacting with oxygen from air producing zinc oxide (ZnO). Another initial corrosion product is zinc hydroxide (Zn(OH)_2_), which forms when moisture is present. Subsequently, ZnO and Zn(OH)_2_ then react over time with carbon dioxide (CO_2_) forming zinc hydroxycarbonates. After several years of exposure to the atmosphere when no other contaminants are present, the patina layer consists mostly of a type of zinc hydroxycarbonate called hydrozincite (Zn_5_(CO_3_)_2_(OH)_6_) [1,6,7]. The formed patina enhances paint adhesion by promoting chemical bonding and increasing the roughness of the HDG surface, thus creating anchorage points [5,8]. Waiting for the natural patina to form on HDG is used as a pretreatment before coating the surface to provide better adhesion properties [4]. A disadvantage of the traditional weathering process is that the formation of a fully developed patina layer can take from eight months to two years’ time for it to be ready for painting [4]. Consequently, there may be a great interest in industry for the possibility to produce an artificial patina of zinc using a fast and versatile method described in our study.

According to the literature, zinc hydroxycarbonates with similar composition to those corrosion products found in naturally forming patinas are possible to be transformed into ZnO nanostructures simply by calcination [9,10]. This opens other interesting applications for our method to alter zinc surfaces using supercritical carbon dioxide (scCO_2_), i.e., the possibility to produce photocatalytic nanostructured ZnO. The scCO_2_ treatment is fast and uses no harmful chemicals compared to conventional hydrothermal synthesis techniques commonly used for ZnO nanowire synthesis [11]. These nanostructures of zinc oxide have been used in high-end technological applications such as dye-sensitized solar cells [12,13,14], piezoelectrics [15] and different types of sensors [16,17]. Furthermore, similar nanowire structures might be used in flexible energy storage systems, supercapacitors, solar and hybrid devices [18,19,20,21,22,23].

Processing with scCO_2_ has been of great interest in materials research because of its green processing properties and wide range of applicability to various processes. It is used to produce many nanostructures, including nanowires, using different kinds of approaches for the nanostructure synthesis [24,25,26,27]. The corrosive behaviour of scCO_2_ on metals has been studied previously. When scCO_2_ and water (H_2_O) are in contact with steel, they react together by forming a compact and protective iron carbonate layer on the steel surface [28,29].

This study presents a rapid and an environmentally friendly method utilizing scCO_2_ for producing an artificial patina layer on a HDG surface. This scCO_2_ treatment utilizes only two simple reactants: CO_2_ and H_2_O.

## 2. Results and Discussion

### 2.1. SEM Imaging and EDS Analysis

**Figure 1:**
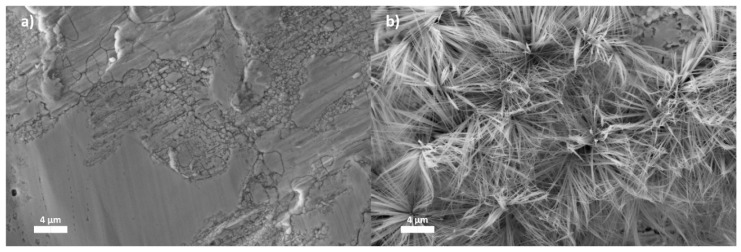
SEM images of rolled hot-dip galvanized (HDG) surface (**a**) and scCO_2_-treated surface (**b**).

We can see a significant change in the surface structure between an untreated HDG surface (Figure 1a) and the scCO_2_-treated sample (Figure 1b). The scCO_2_ treatment produced thin and long nanowires. The nanowires grow mostly straight from the surface but a few seem to have curved towards the tip of the nanowire. Minor branching of the nanowires can be seen as well. The nanowires seem to grow in many angles from single nucleation points. These nucleation points may be caused by pitting type corrosion. In these conditions, the local zinc corrosion around the pits create favourable conditions for nanowire growth as previously suggested in a study by Miles and Mattia [9]. The growth angle from the nucleation points vary from more perpendicular to almost parallel to the surface. Consequently, the overall nanowire growth results in formation of flower-like shapes. Structures with similar morphology and composition have been formed on zinc in previous studies where zinc surface was anodized in the presence of carbonate-containing electrolytes [9,10]. The underlying substrate surface can be seen through the nanowires, which indicates that the surface is not completely covered by the structure.

The energy dispersive spectrometer (EDS) results show that on the reference surface (without the scCO_2_ treatment), the elemental composition was: 8.0 at % oxygen, 2.2 at % aluminum and 89.8 at % zinc. The nanowire structure contained 15.9 at % carbon, 62.9 at % oxygen, 0.1 at % aluminum and 21.1 at % zinc. The amounts of carbon and oxygen present in the nanowire structure could indicate the presence of zinc hydroxycarbonate. The EDS analysis also shows that both the reference zinc surface and the scCO_2_-treated surface contain trace amounts of aluminum. The aluminum from the galvanizing bath reacts with oxygen in air to form aluminum oxide (Al_2_O_3_) on the surface of the HDG sheet [30]. Residual aluminum can also be detected after alkaline cleaning treatment [31]. The presence of aluminum in the scCO_2_ treated sample confirms the uneven surface coverage by the nanowire structure.

### 2.2. FTIR Measurements

**Figure 2:**
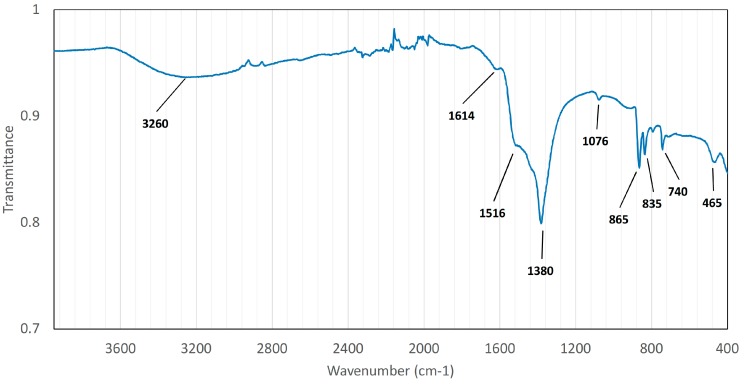
FTIR spectrum measured directly from the sample surface containing the nanowire structure.

The Fourier transform infrared spectroscopy (FTIR) spectrum of the zinc surface is presented in Figure 2. A broad absorption peak can be seen at 3260 cm^−1^ which refers to OH stretching vibration, indicating the presence of hydroxyl groups in the structure [32,33]. The presence of hydroxyl groups is supported by a small band at 1614 cm^−1^ which is attributed to interlayer H_2_O bending [34]. Intense double peak at 1516 cm^−1^ and 1380 cm^−1^ is an indication of antisymmetric ν_3_ stretching modes of carbonates [6,33]. This double peak is typical for zinc hydroxycarbonates containing both carbonate and hydroxyl groups, e.g., hydrozincite [35]. Peak at 1076 cm^−1^ is attributed to ν_1_ stretching of carbonates and is usually inactive in IR-measurements [33,35,36]. However, due to distortion-caused reduction in symmetry of the carbonate this band can become IR-active [33]. Further indication of carbonate presence is due to peaks 865 cm^−1^, 835 cm^−1^ and 740 cm^−1^, which are attributed to carbonate bending modes of ν_2_, out-of-plane ν_2_ and ν_4_, respectively [32,33,36]. Hales et al. stated that the presence of multiple ν_2_ modes is also an indication of symmetry reduction of the carbonate anion, which coheres well with presence of the 1076 cm^−1^ peak. Finally, a small absorption peak at 465 cm^−1^ can be seen as well, which is an indication of some presence of zinc oxide [37,38]. Moreover, in a study where hydrozincite was synthesized from ZnO, water and CO_2_, a similar peak can be found which was attributed to zinc oxide [39]. Since the nanowire structure does not cover the whole surface according to Figure 1b, it is likely that the presence of ZnO is measured from the sample surface rather than the nanowire structure.

### 2.3. XPS Measurements

**Figure 3:**
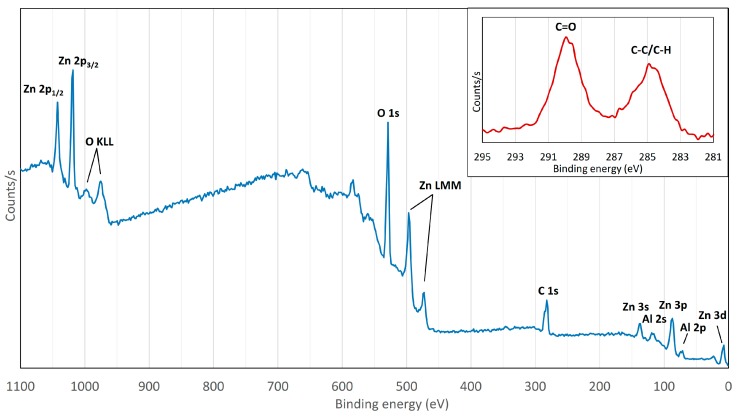
XPS survey spectrum of the scCO_2_-treated sample surface. The spectrum of the C 1s peak is presented in the insert after the sample had gone through mild sputtering.

The XPS survey spectrum of the nanowire-containing surface can be seen from Figure 3. Zinc presence can be seen from Zn 2p_1/2_, Zn 2p_3/2_, Zn 3s and Zn LMM peaks. Clear indication of oxygen presence can be concluded from peaks O 1s, O KLL and carbon presence from peak C 1s. In addition, the surface contains also aluminum due to peaks Al 2s and Al 2p. These results are in good agreement with the EDS results.

The oxygen content was calculated from the X-ray photoelectron spectroscopy (XPS) data from three different points and it was on average 55.3 at %. A minor ZnO presence can be concluded from the FTIR results as well as Al_2_O_3_ presence from the XPS results. However, it can be said that these alone cannot explain the high amount of oxygen on the surface. Therefore, it is expected that the origin of the measured oxygen is derived from the nanowire structure in a form of a zinc hydroxycarbonate.

The insert in Figure 3 shows the C 1s peak. The surface had gone through mild sputtering prior to the analysis in order to confirm that the detected carbon was not present solely as an impurity. We can see two separate peaks at 289.9 eV and 285.0 eV. The latter peak is assigned to C–H and C–C bonding originating from adventitious carbon and, therefore, it is not part of the nanowire structure [31,40,41]. However, the peak at 289.9 eV indicates the presence of carbonate groups in the structure. [31,41]. The peak position is typical for zinc hydroxycarbonates, which is in accordance with the FTIR results [9].

The FTIR and XPS results are in agreement that the nanowire structure contains both hydroxyl and carbonate groups, which agrees with zinc hydroxycarbonate substance. This is supported also by the EDS results. The FTIR spectrum fits well with hydrozincite, a type of zinc hydroxycarbonate, according to earlier studies [33,39]. However, Jambor et al. has stated that the FTIR spectrum is near identical for different zinc hydroxycarbonates exhibiting slightly different stoichiometric compositions [35]. Therefore, a more detailed analysis of the exact composition will be carried out in forthcoming studies.

All in all, the reaction in the scCO_2_ treatment involves HDG surface, H_2_O and scCO_2_. Zinc surface forms ZnO already in atmosphere by reacting with oxygen [42]. It is commonly known that ZnO acts as an intermediate reaction product for the formation of zinc hydroxycarbonates in atmospheric corrosion [1,33]. Moreover, ZnO has also been used as a starting material in hydrozincite powder synthesis together with H_2_O and pressurized CO_2_ [39]. H_2_O can react directly with zinc [1] or with ZnO [42] to produce Zn(OH)_2_. Zn(OH)_2_ is easily dissolved into H_2_O and so it can further react with CO_2_ to produce zinc hydroxycarbonates [42]. However, it has also been proposed that Zn(OH)_2_ can dehydrate back into ZnO and then react with H_2_O and CO_2_ to produce zinc hydroxycarbonates [1]. When CO_2_ dissolves into H_2_O it produces carbonic acid (H_2_CO_3_) that dissociates into HCO_3_^−^ and CO_3_^2−^ ions. The concentration of HCO_3_^−^ is far greater than CO_3_^2−^ in the treatment conditions used, which is why HCO_3_^−^ is more likely to be involved in the formation of the zinc hydroxycarbonate [28]. This is because HCO_3_^−^ is unlikely to dissociate in acidic conditions due to its high pK_a_ of over 10 [43]. Moreover, in earlier studies it has been concluded that the HCO_3_^−^ ions are responsible for the zinc hydroxycarbonate formation [9,44]. To the best of our knowledge, formation of similar nanowire structures in atmospheric corrosion of zinc has not been reported in literature. Factors influencing the formation of the seemingly crystalline structure can be affected by much higher treatment pressure and CO_2_ concentration compared to atmospheric conditions. This is supported by earlier studies where partial pressure has been noticed to affect the formation of zinc corrosion products [33,45].

## 3. Materials and Methods

### 3.1. Materials

A rolled HDG steel sheet with a coating mass of 275 g Zn/m^2^ provided by SSAB Europe Oy was used as a substrate material. The zinc layer was >99% pure with small amounts of alloying elements, e.g., aluminum. The sample dimensions were 25 mm width, 50 mm height and 0.5 mm thickness. The sample was cleaned using an alkaline treatment (Gardoclean 338, Chemetall, Frankfurt, Germany) to remove aluminum oxide layer from the outer surface. Only deionized water, with a conductivity of 2–10 µS, and carbon dioxide (≥97%, AGA, Espoo, Finland) were used for the scCO_2_ treatment.

### 3.2. ScCO_2_ Apparatus and Sample Preparation

**Figure 4:**
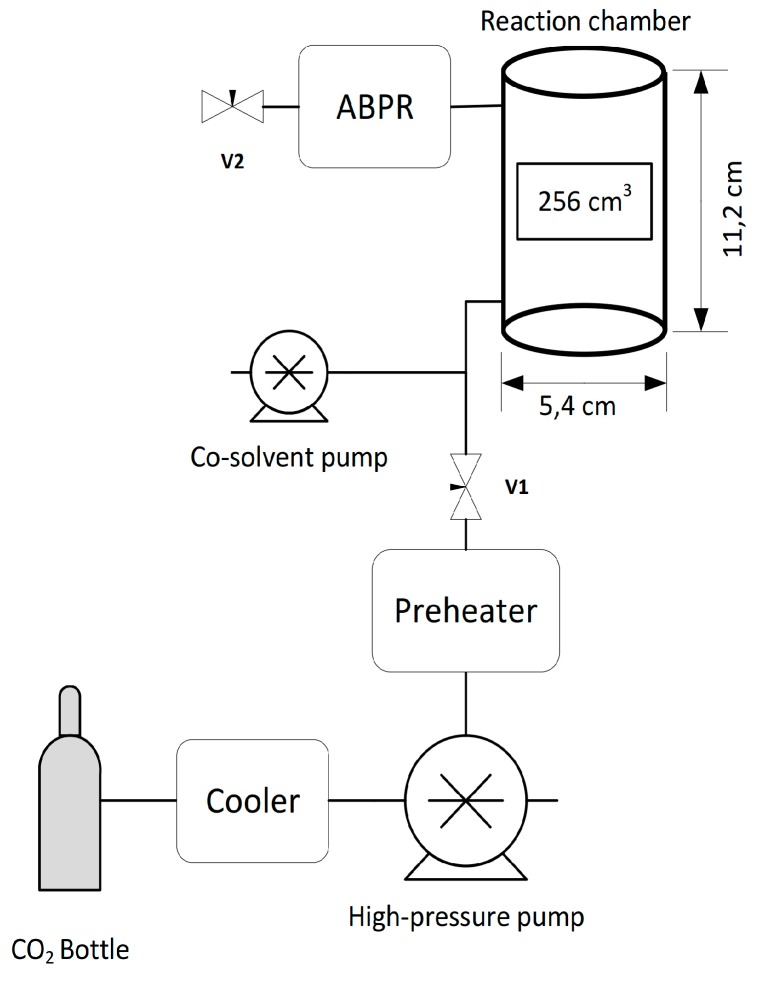
Schematic presentation of the scCO_2_ apparatus.

The scCO_2_ treatments were performed using a Thar Technologies Inc. (Pittsburgh, PA, USA) RESS 250 system. The schematic of the system is presented in Figure 4. The CO_2_ flows initially from a siphon-tubed bottle. The CO_2_ is then cooled by a circulating cooling system to keep it in a liquid state so that it can be pumped into the reaction chamber by a PC-controlled high-pressure piston pump. Before the CO_2_ enters the reaction chamber, it is preheated to gaseous or supercritical state. The reaction chamber is made of 316SS steel and it has an internal heating system built into the walls. The co-solvent pump is used to pump co-solvents during experiments in high-pressures. The PC-controlled automatic back-pressure regulator (ABPR) controls the depressurization rate of the CO_2_ before it flows out from the top of the reaction chamber. Needle valves (V1 and V2) are used to control the pressure inside the system.

The sample was prepared in the following manner. The HDG sheet was placed into the reaction chamber on a sample holder and the valve V2 was closed to ensure that no pressure drop occurred during the treatment. Then the chamber was closed and filled with CO_2_ after which it was heated to 50 °C and pressurized to 300 bar pressure. Valve V1 was then closed and 5 mL of deionized water was introduced to the chamber using the co-solvent pump. The duration of the treatment was 60 min. When the treatment had finished, the valve V2 was opened and the reaction chamber was carefully depressurized of CO_2_ during 10 min using the ABPR.

### 3.3. Characterization Tehcniques

The sample surface was characterized with a field emission scanning electron microscope (FE-SEM, ZEISS ultra plus, Jena, Germany) with energy dispersive spectrometer (EDS, Oxford Instruments INCA Energy 350, Abingdon, UK) using a 15.0 kV acceleration voltage. The EDS results were taken as average values from three separate points on the sample surfaces. FTIR (Bruker Tensor 27, Billerica, MA, USA) with attenuated total reflection (ATR) diamond sample holder and DLaTGS detector was used to analyse the composition of the nanowires in the spectral range of 3950–400 cm^−1^. The FTIR measurements were done directly from the sample surface. X-ray photoelectron spectroscope (XPS) (PHI Quantum 2000, Chanhassen, MN, USA) was used for the surface elemental analysis. A monochromated Al Kα beam (50 W, 15 kV) with a 200 µm spot size was used to obtain the C 1s spectrum. Mild sputtering of the sample surface was performed with Ar^+^ ions. The C 1s peak at 285.0 eV was used for the charge-shift correction.

## 4. Conclusions

A rapid formation of a nanowire structure was obtained on a HDG sheet using only supercritical carbon dioxide and water. The SEM imaging showed that the nanowires had a needle-like shape and they grew from distinct nucleation points on the surface into flower-like structures. The formation of the nanowires was due to a reaction between the zinc surface, water and carbon dioxide. According to the FTIR and XPS analysis, the nanowire structure consisted of hydroxyl and carbonate groups, which is also supported by the EDS results. Therefore, the nanowires are most likely a type of zinc hydroxycarbonate with a proposed stoichiometric formula of Zn*_x_*(CO_3_)*_y_*(OH)*_z_*. Determination of the exact composition as well as the reaction path remains a task for subsequent studies. The method presented in this study shows a great potential for its use as a pretreatment before coating to enhance the paint adhesion and electrochemical properties of the zinc/coating interface. Additionally, the nanowire structure may be used as starting material to produce a ZnO structure that could be used in high-end technological applications, e.g., sensors and dye-sensitized solar cells.
